# Breed dependent regulatory mechanisms of beneficial and non-beneficial fatty acid profiles in subcutaneous adipose tissue in cattle with divergent feed efficiency

**DOI:** 10.1038/s41598-022-08572-8

**Published:** 2022-03-17

**Authors:** Mi Zhou, Zhi Zhu, Hui-Zeng Sun, Ke Zhao, Mike E. R. Dugan, Heather Bruce, Carolyn Fitzsimmons, Changxi Li, Le Luo Guan

**Affiliations:** 1 Department of Agricultural, Food and Nutritional Science, University of Alberta, Edmonton, AB T6G 2P5 Canada; 2 College of Animal Science and Technology, Southwest University, Chongqing, 402460 China; 3 Institute of Dairy Science, Ministry of Education Key Laboratory of Molecular Animal Nutrition, College of Animal Sciences, Zhejiang University, Hangzhou, 310058 China; 4 College of Food Engineering and Nutritional Science, Shaanxi Normal University, Xi’an, Shaanxi, 710119 China; 5 Agriculture and Agri-Food Canada, Lacombe Research and Development Centre, 6000 C & E Trail, Lacombe, AB T4L 1W1 Canada

**Keywords:** Lipids, Metabolomics

## Abstract

The current study aimed to determine whether breed and feed efficiency affect the molecular mechanisms regulating beneficial and non-beneficial fatty acid profiles in subcutaneous adipose tissue of beef steers. Fatty acid profiling and RNA-Seq based transcriptome analysis were performed on subcutaneous adipose tissues collected from beef steers with three divergent breeds (Angus, ANG, n = 47; Charolais, CHAR, n = 48; Kinsella Composite, KC, n = 48) and different residual feed intake (RFI, a measure of feed efficiency). The comparison of fatty acid profiles showed that KC had higher beneficial FAs compared to the other two breeds. Distinct FA profiles between H-RFIfat and L-RFIfat steers was more obvious for KC steers, where H-RFIfat steers tended to have higher proportion of healthy FAs and lower proportion of the unhealthy FAs. A higher number of differentially expressed (DE) genes were observed for KC steers, whereas ANG and CHAR steers had a lower number of DE genes between H- and L-RFIfat steers. The association analyses of the gene expressions and FA profiles showed that 10 FA metabolism-associated genes together with the one upstream regulator (*SREBF1*) were associated with the proportion of C18:2n-6, total n-6, PUFA and PUFA/SFA for KC steers but not the other two breeds. Subcutaneous adipose tissue FA profiles and healthy FA index differed in cattle with divergent feed efficiency and such variation was unique for the three examined cattle breeds. Key FA metabolism-associated genes together with *SREBF1* which is the upstream regulator of a set of genes involved in lipid metabolism may be of importance for genetic selection of meat with higher healthy FA index in beef cattle.

## Introduction

Public interest in beef fatty acid (FA) composition is increasing because of its influence on human health^1^ and meat quality characteristics including palatability, shelf life, and flavor^2–4^. Notably, high saturated FA (SFA) content in beef, particularly C12:0, C14:0 and C16:0, has been reported to lead to high blood cholesterol, and increased risks for atherosclerosis and coronary heart disease^5,6^. However, monounsaturated FA (MUFA) and polyunsaturated FA (PUFA) can reduce both total cholesterol and low-density lipoprotein cholesterol levels^7,8^. In addition, in cattle, ingested PUFA are biohydrogenated by rumen microbes, and partial biohydrogenation products such as conjugated linoleic acid (CLA) and trans 18:1 isomers can be absorbed and incorporated into tissues^9,10^. The main natural isomer of CLA (*cis(c)*9, *trans(t)*11-CLA) and its precursor *t*11-18:1 have been found to have anti-carcinogenic and anti-atherogenic properties (Schmid et al. 2006), while trans10-18:1 (t10-18:1) has been found to have atherogenic effects in humans^8,11^ and model animals^12^. In recent review papers by Vahmani et al*.*^13^ and Pethick et al*.*^14^, the impact of meat-derived FAs on human health has been extensively discussed, which specifically emphasized the importance of the long-chain n-3 PUFA of the meat products derived from pasture-based systems in lowering the risk of fatal coronary heart disease. Both studies claimed that ruminant meat-derived long-chain n-3 PUFAs are essential for human health. Based on the existing research and cross-studies analyses, extensive research has thus been undertaken to improve the balance of beneficial to non-beneficial FA in beef.

Recent studies have reported that among the 59 FAs identified in beef subcutaneous adipose tissue^15^, six of them were moderately or highly heritable (heritability ≥ 0.4)^16^, suggesting that host genetics play an important role in controlling synthesis of certain FAs. In addition, finishing cattle on a high grain diet (a common practice) increased the t10-18:1 and reduced the t11-18:1 in the adipose tissue compared to those fed with high forage diets^17^, suggesting that the composition of PUFA biohydrogenation intermediates in beef is affected by diet^18^. For those FAs which display low or no heritability, the regulatory mechanisms are largely unknown, and this may be due to them arising from rumen microbial lipid metabolism as the rumen microbiome is associated with FA profiles in subcutaneous adipose tissue^19^.

To date, the underlying molecular mechanisms for determining beef FA profiles, especially the ratio of beneficial to non-beneficial FA, have not been characterized. In this study, we hypothesized that the ratio of beneficial FA (total proportion of 18:3n-3; c9,t11-18:2 and t11-18:1) to non-beneficial FA (total proportion of C14:0; C16:0; and t10-18:1) (defined as healthy FA index) is a trait that can be regulated by gene expression, making it a trait useful for selection in animal breeding programs. To achieve this aim, we performed FA profiling and RNA-Seq-based transcriptome analysis of subcutaneous adipose tissue collected from cattle in three divergent breeds (Angus, Charolais, and Kinsella Composite) with different feed efficiencies. It is known that the amount of subcutaneous adipose tissue affects beef quality and is the priority site for lipogenesis, followed by intramuscular fat deposition^20^. In addition, excess subcutaneous adipose tissue was associated with decreased feed efficiency and depreciated carcass, and animals with higher feed efficiency tended to have less subcutaneous adipose tissue^21,22^. As suggested by Bassarab et al.^23^, subcutaneous adipose tissue (backfat thickness) has now been included to justify residual feed intake (RFI), one of the commonly used feed efficiency traits for the herds used in this study^23^. It has also been reported that low residual feed intake (Low-RFI, more efficient) animals tended to have leaner carcasses, less fat and more protein deposition^24–27^. Therefore, in this study, we assessed the effects of breed and RFI on FA profiles and healthy FA index in subcutaneous adipose tissue collected from 143 steers, and investigated the genome wide expression profiles of subcutaneous adipose tissue to elucidate the underlying molecular mechanisms of adipogenesis and genes associated with the identified variations in FA profiles and healthy FA index.

## Results

### Effect of breed on phenotypic measures

The performance traits from a total 143 beef cattle are presented in Table 1. DMI and ADG of ANG animals were higher than those of CHAR cattle (*p* < 0.05) and subcutaneous adipose tissue thickness of ANG and KC steers was higher compared to that of CHAR steers (*p* < 0.05). Significant differences in marbling score were also found between ANG and KC steers with higher marbling score in ANG (*p* < 0.05).

**Table 1:** Effects of different breeds on performance traits of beef cattle.

Trait*	ANG^1^ (n = 47)	CHAR^2^ (n = 48)	KC^3^ (n = 48)	SEM	*p* value
DMI (kg)	11.46^a^	10.66^b^	10.16^b^	0.120	< .0001
ADG (kg)	1.72^a^	1.62^ab^	1.52^b^	0.024	0.003
RFIfat (kg)	0.18	0.02	− 0.05	0.075	0.459
Backfat thickness (mm)	11.35^a^	8.55^b^	11.44^a^	0.280	< .0001
Marbling score	423.04^a^	404.58^a^	373.37^b^	4.945	0.000

*DMI = dry matter intake; ADG = average daily gain; RFIfat = residual feed intake adjusted for backfat thickness.

^1^Angus breed; ^2^Charolais breed; ^3^Kinsella Composite breed.

Means in the same row with different superscripts (a, b) indicate significance (*p* < 0.05).

### FA profiles among the three cattle breeds

In total, 49 FAs were identified from subcutaneous adipose tissues of the entire sample set, and the proportion of each identified FA is summarized in Table 2. For the beneficial FAs, the proportion of t11-18:1 was higher in CHAR and KC than in ANG *(p* < 0.05). Meanwhile, proportion of c9,t11-CLA was higher in KC than in ANG and CHAR (*p* < 0.05), but no difference was found between ANG and CHAR animals (*p* > 0.05). Mean proportion of C18:3n-3 was lower in ANG when compared to CHAR (*p* < 0.05) and were similar between ANG and KC (*p* < 0.05). Among the non-beneficial FAs, the mean proportions of C16:0 and overall SFA in ANG were higher than those in CHAR and KC (*p* < 0.05), whereas the mean proportions of C12:0 and C14:0 in ANG were lower than those in CHAR (*p* < 0.05). The mean proportion of t10-18:1 in CHAR was higher than that in ANG and KC (*p* < 0.05), and no significant difference was found between ANG and KC. Other long-chain n-3 PUFAs (20:5n-3, 22:5n-3 and 22:6n-3) were below detection limit for the current study among the samples. KC had significant higher healthy FA index (3.33 ± 0.77%, mean ± SD) than in ANG (2.50 ± 0.78%, mean ± SD) and CHAR (2.94 ± 0.77%, mean ± SD) (*p* < 0.01).

**Table 2:** Effects of different breeds on fatty acid proportion (%) in subcutaneous tissues of beef cattle.

Trait*	ANG (n = 47)	CHAR (n = 48)	KC (n = 48)	SEM	*p* value
14:0	3.78^b^	4.16^a^	3.90^ab^	0.056	0.017
16:0	28.39^a^	26.56^b^	27.11^b^	0.172	< 0.0001
18:0	11.80	11.70	11.11	0.024	0.307
SFA	46.03^a^	44.36^b^	43.91^b^	0.277	0.004
BCFA	1.35^b^	1.65^a^	1.39^b^	0.024	< 0.0001
SFA + BCFA	47.38^a^	46.01^b^	45.30^b^	0.286	0.010
*c*9-16:1	4.84b	5.61^a^	5.40^a^	0.095	0.002
*c*9-18:1	37.71^ab^	36.88^b^	38.46^a^	0.224	0.014
*c*11-18:1	1.72^b^	2.01^a^	1.90^ab^	0.040	0.010
*t*10-18:1	1.17^b^	1.47^a^	1.01^b^	0.053	0.001
*trans*-18:1	2.53^b^	3.22^a^	2.54^b^	0.067	< 0.0001
MUFA	50.72^b^	51.66^ab^	52.47^a^	0.281	0.038
ADFA	0.44^b^	0.54^a^	0.53^a^	0.009	< 0.0001
CLA	0.28^b^	0.28^b^	0.43^a^	0.011	< 0.0001
n-6	1.03^c^	1.35^a^	1.11^b^	0.019	< 0.0001
18:3n-3	0.11^b^	0.13^a^	0.12^b^	0.003	0.002
PUFA	1.14^c^	1.48^a^	1.24^b^	0.021	< 0.0001
PUFA/SFA	0.025^c^	0.034^a^	0.028^b^	0.001	< 0.0001
FA ratio	0.025^c^	0.029^b^	0.033^a^	0.001	< 0.0001
SCD proxy	0.27^b^	0.26^b^	0.30^a^	0.004	0.008

*The concentrations of fatty acids were expressed as a percentage of fatty acid methyl esters (FAME) quantified. Significant effects (*p* < 0.05) of breed on fatty acid are presented. *c* = *cis*; *t* = *trans*; SFA (sum of saturated fatty acid) = 10:0 + 12:0 + 14:0 + 15:0 + 16:0 + 17:0 + 18:0 + 19:0 + 20:0; BCFA (sum of branched chain fatty acid) = *iso*−14:0 + *iso*-15:0 + *anteiso*-15:0 + *iso*-16:0 + *iso*-17:0 + *anteiso*-17:0 + *iso*-18:0; SFA + BCFA = sum of SFA and BCFA; *trans-*18:1 (sum of *trans-*18:1) = *t*6-18:1 + *t*9-18:1 + *t*10-18:1 + *t*11-18:1 + *t*12-18:1 + *t*13-/*t*14-18:1; MUFA (sum of monounsaturated fatty acid) = *c*9-14:1 + *c*7-16:1 + *c*9-16:1 + *c*11-16:1 + *c*9-17:1 + *c*9-18:1 + *c*11-18:1 + *c*12-18:1 + *c*13-18:1 + *c*14-18:1 + *t*6-18:1 + *t*9-18:1 + *t*10-18:1 + *t*11-18:1 + *t*12-18:1 + *t*13-/*t*14-18:1 + *c*9-20:1 + *c*11-20:1; ADFA (sum of atypical dienes fatty acid) = *c*9,*t*14-/*c*9,*t*13-18:2 + *c*9,*t*15-18:2 + *c*9,*t*12-18:2 + *t*11,*c*15-18:2 + *c*9,*c*15-18:2; CLA (sum of conjugated linoleic acid) = *t*7,*c*9-18:2 + *c*9,*t*11-18:2 + *t*9,*c*11-18:2 + *t*11,*t*13-18:2 + *t*7,*t*9-/*t*10,*t*12-18:2; n-6 (sum of omega 6 fatty acids) = 18:2n-6 + 20:3n-6 + 20:4n-6; n-3 (sum of omega 3 fatty acids) = 18:3n-3; PUFA (sum of polyunsaturated fatty acid) = 18:2n-6 + 20:3n-6 + 20:4n-6 + 18:3n-3; PUFA/SFA = ratio of PUFA to SFA; n-6/n-3 = ratio of n − 6 to n − 3 PUFA; FA ratio = (18:3n-3 + *c*9,*t*11-18:2 + *t*11-18:1)/(14:0 + 16:0 + *t*10-18:1); *SCD* proxy (stearoyl-CoA desaturase proxy) = *c*9-14:1/(*c*9-14:1 + 14:0).

Means in the same row with different superscripts (a, b, c) indicate significance (*p* < 0.05).

Only major FAs/FA groups are listed in this table. Detailed proportions of the FAs of individual samples are listed in Supplementary Table S6.

### Subcutaneous adipose tissue FA profiles between high- and low- RFI steers

**Figure 1:**
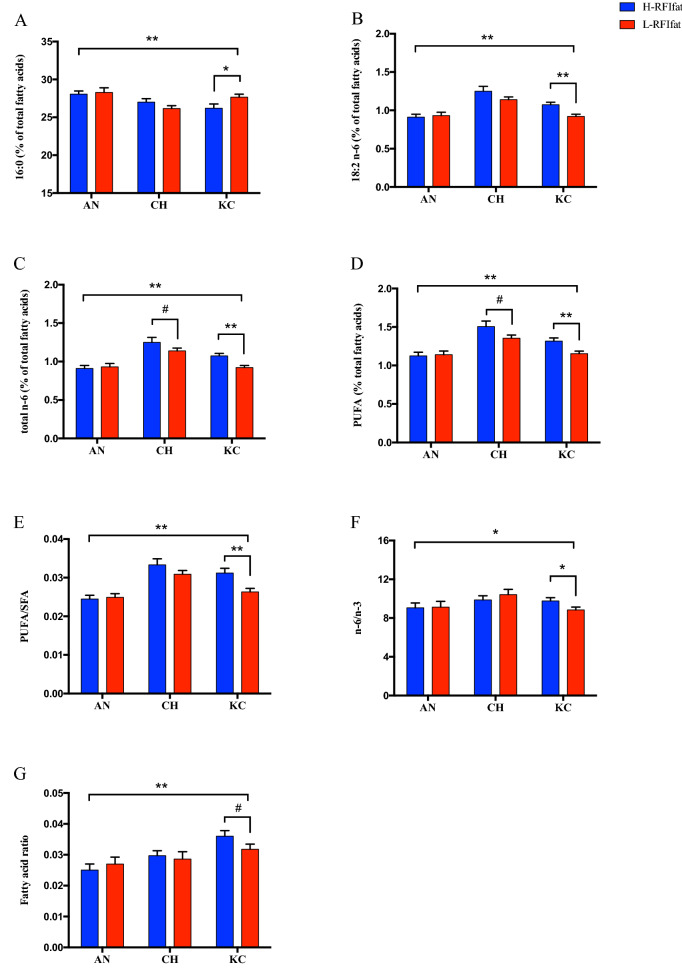
Differential abundant FAs between H-RFI and L-RFI steers as analyzed for each cattle breed respectively. Significance was indicated as follows: **p < 0.01; *0.01 ≤ *p* < 0.05; #0.05 ≤ *p* < 0.1.

Mean subcutaneous adipose tissue thickness and marbling score were similar between H-RFI and L-RFI animals in all three breeds (Table 3). However, the effect of RFI on FA profiles differed among the breeds. As shown in Fig. 1, the proportion of C18:2n-6, total n-6, PUFA, PUFA/SFA and healthy FA index tended to be higher in subcutaneous adipose tissue of H-RFI than those of L-RFI (*p* < 0.01), while C16:0 proportion was lower in subcutaneous adipose tissue of H-RFI than that of L-RFI in KC steers (*p* = 0.029). In addition, total n-6 and PUFA proportions were tentatively higher in subcutaneous adipose tissue of H-RFI than those of L-RFI in CHAR steers. No difference was observed in subcutaneous adipose tissue FA profiles between H-RFI and L-RFI ANG steers. For individual beneficial and non-beneficial FAs, only the C16:0 (non-beneficial) was found to be significantly higher in subcutaneous adipose tissue of L-RFI than that of H-RFI in KC steers (27.67% vs. 26.21%, *p* = 0.029). The healthy FA index was tentatively higher in subcutaneous adipose tissue of H-RFI than that of L-RFI in KC animals (3.60% vs. 3.18%, *p* = 0.093).

**Table 3:** Effects of breed and residual feed intake adjusted for backfat thickness (RFIfat) on fatty acid proportion (%) in subcutaneous tissues and performance traits of beef cattle.

Trait*	ANG^1^			CHAR^2^			KC^3^			SEM	*p* value		
	H-RFIfat^4^ (n = 16)	L-RFIfat^5^ (n = 14)	*p* value^#^	H-RFIfat (n = 17)	L-RFIfat (n = 17)	*p* value^#^	H-RFIfat (n = 18)	L-RFIfat (n = 21)	*p* value^#^		Breed	RFIfat	Breed × RFIfat
14:0	3.75^bc^	3.89^abc^	0.463	4.32^a^	4.13^ab^	0.426	3.66^c^	4.05^abc^	0.126	0.069	0.029	0.405	0.197
16:0	28.07^a^	28.29^a^	0.769	27.01^ab^	26.16^b^	0.156	26.21^b^	27.67^a^	0.029	0.200	0.003	0.466	0.038
18:0	12.13	11.56	0.566	11.98	11.85	0.863	11.02	10.81	0.791	0.240	0.159	0.534	0.933
SFA	45.90^a^	45.79^a^	0.903	45.29^a^	43.99^ab^	0.204	42.64^b^	44.30^ab^	0.209	0.333	0.014	0.895	0.156
BCFA	1.32^c^	1.44^bc^	0.232	1.67^a^	1.61^ab^	0.549	1.43^c^	1.38^c^	0.496	0.028	< .0001	0.928	0.299
SFA + BCFA	47.22^a^	47.23^a^	0.986	46.96^a^	45.60^ab^	0.207	44.06^b^	45.68^ab^	0.237	0.347	0.019	0.894	0.183
*c*9-16:1	4.84^c^	4.93^bc^	0.843	5.51^abc^	5.77^a^	0.523	5.39^abc^	5.66^ab^	0.457	0.116	0.025	0.370	0.936
*c*9-18:1	38.01^ab^	37.69^ab^	0.720	36.25^b^	37.48^b^	0.162	39.29^a^	37.97^ab^	0.186	0.274	0.024	0.804	0.134
*c*11-18:1	1.69	1.67	0.908	1.88	1.99	0.485	1.98	1.87	0.549	0.049	0.072	0.953	0.615
*t*10-18:1	1.11^ab^	1.09^ab^	0.913	1.29^a^	1.33^a^	0.804	1.06^ab^	0.95^b^	0.456	0.050	0.039	0.767	0.790
*trans*-18:1	2.48^c^	2.51^c^	0.889	3.08^a^	3.05^ab^	0.880	2.64^bc^	2.44^c^	0.337	0.069	0.001	0.604	0.744
MUFA	50.91^b^	50.86^b^	0.963	50.72^b^	52.21^ab^	0.169	53.56^a^	52.17^ab^	0.301	0.343	0.049	0.982	0.207
ADFA	0.44^b^	0.45^b^	0.822	0.54^a^	0.53^a^	0.798	0.55^a^	0.53^a^	0.364	0.010	< .0001	0.607	0.757
CLA	0.27^b^	0.28^b^	0.902	0.23^b^	0.27^b^	0.413	0.47^a^	0.43^a^	0.210	0.014	< .0001	0.925	0.418
n-6	1.01^c^	1.02^c^	0.798	1.36^a^	1.23^b^	0.097	1.19^b^	1.03^c^	0.002	0.022	< .0001	0.012	0.122
18:3n-3	0.12^b^	0.12^b^	0.969	0.14^a^	0.12^ab^	0.107	0.13^ab^	0.12^b^	0.446	0.003	0.145	0.195	0.543
PUFA	1.13^c^	1.14^c^	0.828	1.51^a^	1.36^b^	0.082	1.32^b^	1.15^c^	0.003	0.023	< .0001	0.013	0.140
PUFA/SFA	0.024^b^	0.025^b^	0.778	0.033^a^	0.031^a^	0.198	0.031^a^	0.026^b^	0.003	0.001	< .0001	0.015	0.074
n-6/n-3	9.06^b^	9.13^ab^	0.928	9.88^ab^	10.42^a^	0.435	9.77^ab^	8.83^b^	0.042	0.184	0.049	0.765	0.212
FA ratio	0.025^c^	0.027^bc^	0.512	0.030^bc^	0.029^bc^	0.687	0.036^a^	0.031^ab^	0.093	0.001	0.0004	0.479	0.288
SCD proxy	0.27^ab^	0.28^ab^	0.647	0.27^b^	0.26^b^	0.912	0.30^a^	0.30^a^	0.999	0.005	0.013	0.841	0.924
DMI (kg)	12.49^a^	10.73^c^	0.001	11.87^ab^	9.83^d^	< .0001	11.29^bc^	9.24^d^	< .0001	0.156	< .0001	< .0001	0.826
ADG (kg)	1.62^ab^	1.79^a^	0.132	1.65^ab^	1.62^ab^	0.661	1.54^b^	1.51^b^	0.762	0.027	0.021	0.465	0.206
Backfat thickness (mm)	11.13^a^	11.28^a^	0.899	8.06^b^	8.25^b^	0.830	10.74^a^	11.93^a^	0.271	0.332	< .0001	0.397	0.720
Marbling score	433.33^a^	409.29^ab^	0.257	400.00^abc^	381.18^bc^	0.331	380.33^bc^	367.37^c^	0.370	5.520	0.002	0.078	0.912

*c = *cis*; *t* = *trans*; SFA (sum of saturated fatty acid) = 10:0 + 12:0 + 14:0 + 15:0 + 16:0 + 17:0 + 18:0 + 19:0 + 20:0; BCFA (sum of branched chain fatty acid) = *iso*-14:0 + *iso*-15:0 + *anteiso*-15:0 + *iso*-16:0 + *iso*-17:0 + *anteiso*-17:0 + *iso*-18:0; SFA + BCFA = sum of SFA and BCFA; *trans-*18:1 (sum of *trans-*18:1) = *t*6-18:1 + *t*9-18:1 + *t*10-18:1 + *t*11-18:1 + *t*12-18:1 + *t*13-/*t*14-18:1; MUFA (sum of monounsaturated fatty acid) = *c*9-14:1 + *c*7-16:1 + *c*9-16:1 + *c*11-16:1 + *c*9-17:1 + *c*9-18:1 + *c*11-18:1 + *c*12-18:1 + *c*13-18:1 + *c*14-18:1 + *t*6-18:1 + *t*9-18:1 + *t*10-18:1 + *t*11-18:1 + *t*12-18:1 + *t*13-/*t*14-18:1 + *c*9-20:1 + *c*11-20:1; ADFA (sum of atypical dienes fatty acid) = *c*9,*t*14-/*c*9,*t*13-18:2 + *c*9,*t*15-18:2 + *c*9,*t*12-18:2 + *t*11,*c*15-18:2 + *c*9,*c*15-18:2; CLA (sum of conjugated linoleic acid) = *t*7,*c*9-18:2 + *c*9,*t*11-18:2 + *t*9,*c*11-18:2 + *t*11,*t*13-18:2 + *t*7,*t*9-/*t*10,*t*12-18:2; n-6 (sum of omega 6 fatty acids) = 18:2n-6 + 20:3n-6 + 20:4n-6; n-3 (sum of omega 3 fatty acids) = 18:3n-3; PUFA (sum of polyunsaturated fatty acid) = 18:2n-6 + 20:3n-6 + 20:4n-6 + 18:3n-3; PUFA/SFA = ratio of PUFA to SFA; n-6/n-3 = ratio of n − 6 to n − 3 PUFA; FA ratio = (18:3n-3 + *c*9,*t*11-18:2 + *t*11-18:1)/(14:0 + 16:0 + *t*10-18:1); *SCD* proxy (stearoyl-CoA desaturase proxy) = *c*9-14:1/(*c*9-14:1 + 14:0); DMI = dry matter intake; ADG = average daily gain.

^#^Statistical analysis was performed within each breed.

^1^Angus breed; ^2^Charolais breed; ^3^Kinsella Composite breed; ^4^High RFIfat steers (RFIfat > 0.5); ^5^Low RFIfat steers (RFIfat < − 0.5).

Means in the same row with different superscripts (a, b, c, d) indicate significance (*p* < 0.05).

Only major FAs/FA groups are listed in this table.

### Correlation between RFI and FA profiles

**Figure 2:**
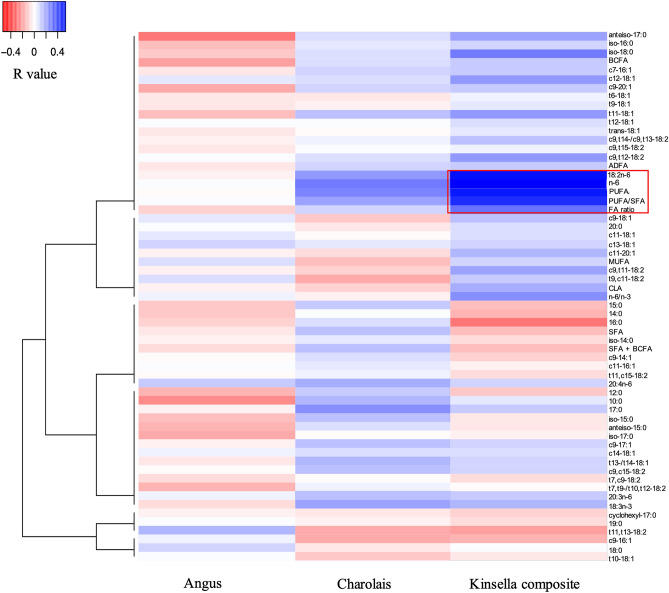
Correlations between FA profiles and RFI for the three breeds. The correlations of statistical significance (*p* < 0.05) identified from the KC breed were highlighted in red square.

Correlation analysis was performed to identify the relationship between RFI and FA proportions. Low correlations (r ranged from 0.16 to 0.19) were found between RFI and n-6 FAs (including 18:2n-6 and 20:3n-6), PUFA and PUFA/SFA, as well as marbling score, when analyzing the entire dataset without separating breeds (data not shown). When the correlation analysis was performed within each breed, moderate positive correlations were observed between RFI and 18:2n-6 (r = 0.54), total n-6 (r = 0.51), PUFA (r = 0.50) and PUFA/SFA (r = 0.46) in the KC group (Fig. 2). RFI was also positively correlated with healthy FA index in KC animals (r = 0.29, *p* = 0.048). No significant correlation between RFI and healthy FA index was found in ANG and CHAR steers (*p* > 0.05).

### Identification of bovine subcutaneous adipose tissue transcriptome

To further understand gene expression profiles and their relations with RFI, 22 steers with different RFI were selected for transcriptome analysis using RNA-Seq, including 8 steers of ANG (4 H-RFI, 1.17 ± 0.36; 4 L-RFI, − 0.99 ± 0.17; *p* < 0.001), 6 of CHAR (3 H-RFI, 0.84 ± 0.22; 3 L-RFI, − 1.00 ± 0.03; *p* < 0.01), and 8 of KC (4 H-RFI, 1.28 ± 0.67; 4 L-RFI, − 1.33 ± 0.43; *p* < 0.01). In total, 24,692,450 ± 3,237,147 clean reads were aligned to the reference bovine genome, which identified 21,057 genes with 13,531 ± 52, 13,616 ± 106 and 13,634 ± 102 expressed in ANG, CHAR and KC, respectively. Among all of the identified genes, 13,125 genes were commonly expressed in subcutaneous adipose tissue of all steers; while 48, 77, and 160 genes were exclusively expressed for ANG, CHAR and KC respectively. Further DE analysis identified 46, 39 and 177 genes were differentially expressed between H-RFI and L-RFI groups for ANG, CHAR and KC steers, respectively (Supplementary Table S1). Among the DE genes, 42, 22, 103 were down-regulated in adipose tissue of L-RFI steers when compared to H-RFI steers of ANG, CHAR and KC, respectively (Supplementary Table S1). In addition, all of the up-regulated DE genes were breed-exclusive (Supplementary Fig. S2); whereas only one down-regulated DE gene, *SMPD3* (sphingomyelin phosphodiestrase 3) was shared by the three breeds (log_2_FC = − 2.401, − 3.285, and − 1.367 in ANG, CHAR, and KC, respectively).

### Functional analysis of commonly expressed genes among three breeds and DE genes between H- and L-RFI steers within each breed

**Figure 3:**
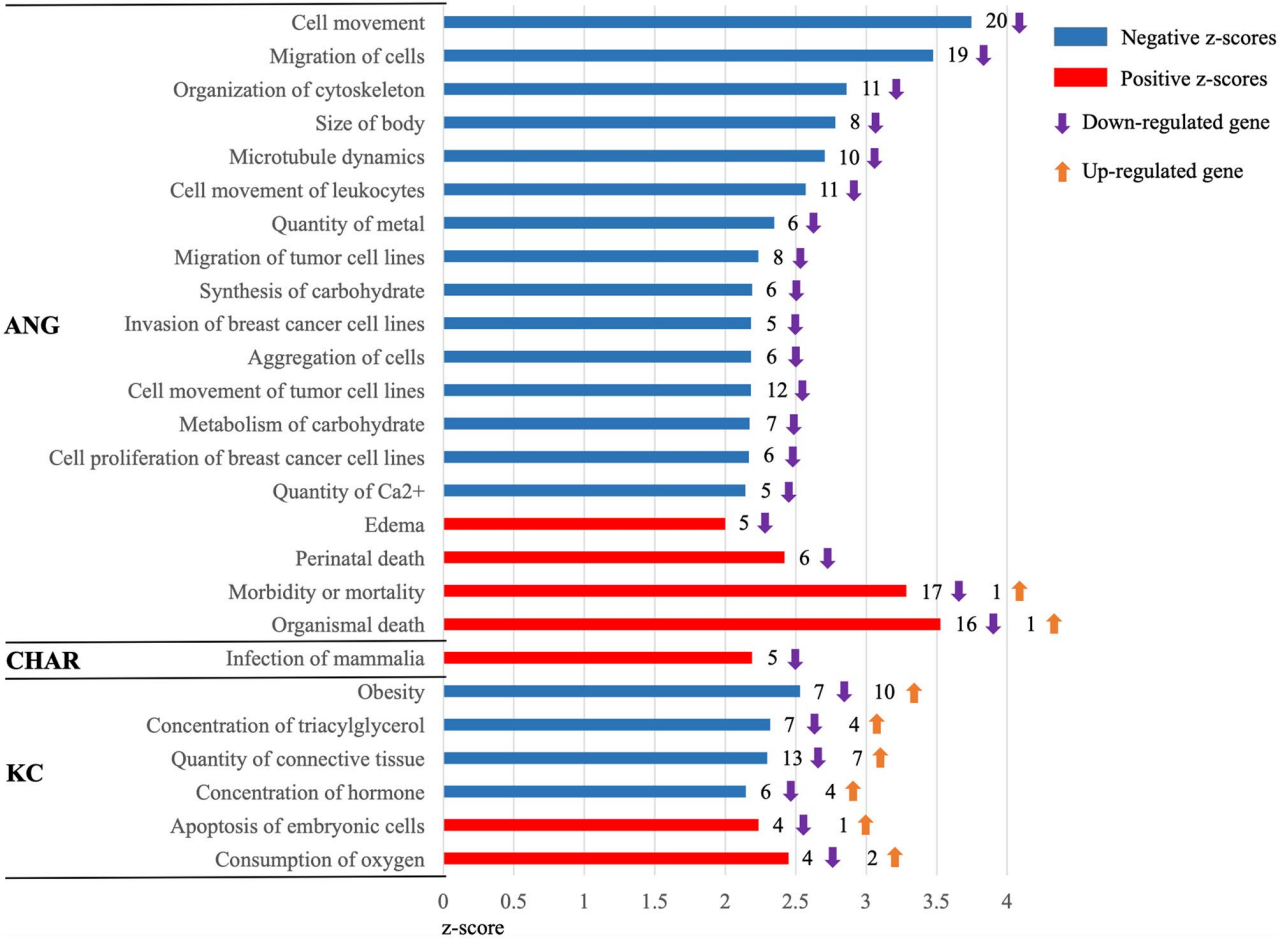
Functional classification of differentially expressed genes using IPA (Ingenuity Pathway Analysis) in different breeds of beef cattle. Positive z-scores are indicated with red bars and negative z-scores with blue bars. A positive z-score (2) indicates an increased predicted activation state, while a negative z-score (− 2) indicates a reduction in function. The number to the right of the bar is the number of differentially expressed (DE) genes. Up arrow and down arrow represent the DE genes up- and down-regulated in L-RFIfat compared to H-RFIfat steers, respectively. ANG, Angus; CHAR, Charolais; KC, Kinsella Composite.

A total of 2,569 GO terms were generated with 1,182 GO terms significantly enriched (*p* < 0.001) from the commonly expressed genes (Supplementary Table S2). In the molecular function category, most of the commonly expressed genes were involved in binding (39.8%) and catalytic activity (39.2%). Cellular process (30.7%), metabolic process (24.0%), cellular component organization or biogenesis (8.6%), biological regulation (8.5%) and localization (7.5%) were the predominant GO terms in biological process category. A high proportion of commonly expressed genes were assigned to the GO terms of cell part (39.5%), organelle (28.2%), macromolecular complexes (16.0%) and membrane (11.9%) in the cellular component category. No function was enriched for DE genes based on PANTHER and DAVID bioinformatic tools. When the DE genes were subjected to IPA analysis, 19, 1 and 6 enriched functions involved in 11, 1 and 6 functional categories (z-score ≥ 2 or z-score ≤ − 2) were identified for ANG, CHAR and KC animals, respectively (Fig. 3).

Detailed list of the DE genes involved in these enriched functions are listed in Table 4, among which only one GO term was associated with lipid metabolism. In the adipose tissue of KC L-RFI steers, 4 up-regulated genes (*DLK1*, *LEPR*, *TLR5*, *PDK4*) and 7 down-regulated genes (*ACLY*, *CIDEA*, *G0S2*, *LDLR*, *MFSD2A*, *SERPINE1*, *SREBF1*) involved in lipid metabolism and were associated with lower triacylglycerol concentration (z-score =  − 2.319) (Table 5). Further upstream analysis using IPA identified 11, 9 and 78 upstream regulators (z-score ≥ 2 or z-score ≤ − 2 Supplementary Table S3) targeting DE genes in ANG, CHAR and KC, respectively. Among them *SREBF1*, a DE gene identified from KC steers, was found to be an upstream regulator that inhibited 11 target molecules (z-score =  − 2.78), including 3 up-regulated DE genes (*DLK1*, *TF*, *TTN*) and 8 down-regulated DE genes (*AACS*, *ACL*Y, *CIDEA*, *LDLR*, *LSS*, *SERPINE1*, *SQLE*, *TM7SF2*) in L-RFI as compared to H-RFI KC steers (Supplementary Table S4).

**Table 4:** GO term enrichment results of all differentially expressed genes using IPA within each breed.

Breed	Category	Module	Functions	z-score^†^	*Adjusted p* value	Predicted action	Involved genes
ANG	Organismal survival	17	Organismal death	3.527	0.000	Activated	*BMP7*,*CFB*,*CXCL14*,*EPHB2*,*HAS2*,*ITGA11*,*MYB*,*PDPN*,*PERP*,*PLAU*,*PTHLH*,*ROR1*,*SFRP4*,*SYCP3*,*TH*,*THBD*,*WNT2*
		18	Morbidity or mortality	3.285	0.000	Activated	*BMP7*,*CFB*,*CXCL14*,*EPHB2*,*HAS2*,*ITGA11*,*MYB*,*PDPN*,*PERP*,*PLAU*,*PTHLH*,*RASSF5*,*ROR1*,*SFRP4*,*SYCP3*,*TH*,*THBD*,*WNT2*
		6	Perinatal death	2.421	0.005	Activated	*BMP7*,*CXCL14*,*PDPN*,*PTHLH*,*TH*,*WNT2*
	Organismal injury and abnormalities	5	Edema	2.000	0.003	Activated	*HAS2*,*PDPN*,*PLAU*,*ROR1*,*TNFAIP6*
	Cell signaling, molecular transport, vitamin and mineral metabolism	5	Quantity of Ca^2+^	−2.143	0.004	Inhibited	*ACKR2*,*NTS*,*PLAU*,*PTHLH*,*TPSAB1/TPSB2*
	Cellular development, cellular growth and proliferation	6	Cell proliferation of breast cancer cell lines	−2.168	0.002	Inhibited	*BMP7*,*HAS2*,*MYB*,*PLAU*,*PTHLH*,*SMPD3*
	Carbohydrate metabolism	7	Metabolism of carbohydrate	−2.173	0.001	Inhibited	*BMP7*,*GFPT2*,*HAS2*,*NTS*,*PLAU*,*THBD*,*TNFAIP6*
		6	Synthesis of carbohydrate	−2.191	0.001	Inhibited	*BMP7*,*HAS2*,*NTS*,*PLAU*,*THBD*,*TNFAIP6*
	Cellular movement	12	Cell movement of tumor cell lines	−2.182	0.000	Inhibited	*BMP7*,*CXCL14*,*EPHB2*,*HAS2*,*MYB*,*PDPN*,*PLAU*,*PTHLH*,*ROR1*,*SFRP4*,*THBD*,*TIAM1*
		5	Invasion of breast cancer cell lines	−2.183	0.001	Inhibited	*HAS2*,*MYB*,*PLAU*,*ROR1*,*TIAM1*
		8	Migration of tumor cell lines	−2.235	0.001	Inhibited	*EPHB2*,*HAS2*,*PDPN*,*PLAU*,*PTHLH*,*ROR1*,*SFRP4*,*TIAM1*
		19	Migration of cells	−3.475	0.000	Inhibited	*ACKR2*,*BMP7*,*CFB*,*CXCL14*,*EPHB2*,*HAS2*,*HDC*,*MYB*,*NTS*,*PDPN*,*PLAU*,*PTHLH*,*RASSF5*,*ROR1*,*SFRP4*,*THBD*,*TIAM1*,*TNFAIP6*,*TPSAB1/TPSB2*
		20	Cell movement	−3.747	0.000	Inhibited	*ACKR2*,*BMP7*,*CFB*,*CXCL14*,*EPHB2*,*HAS2*,*HDC*,*ITGA11*,*MYB*,*NTS*,*PDPN*,*PLAU*,*PTHLH*,*RASSF5*,*ROR1*,*SFRP4*,*THBD*,*TIAM1*,*TNFAIP6*,*TPSAB1/TPSB2*
	Cell-to-cell signaling and interaction	6	Aggregation of cells	−2.183	0.000	Inhibited	*BMP7*,*HDC*,*PDPN*,*PTHLH*,*THBD*,*WNT2*
	Molecular transport	6	Quantity of metal	−2.348	0.002	Inhibited	*ACKR2*,*AP3B2*,*NTS*,*PLAU*,*PTHLH*,*TPSAB1/TPSB2*
	Cellular movement, hematological system development and function, immune cell trafficking	11	Cell movement of leukocytes	−2.571	0.000	Inhibited	*ACKR2*,*CFB*,*CXCL14*,*HDC*,*MYB*,*PDPN*,*PLAU*,*RASSF5*,*THBD*,*TIAM1*,*TPSAB1/TPSB2*
	Cellular assembly and organization, cellular function and maintenance	10	Microtubule dynamics	−2.706	0.002	Inhibited	*BMP7*,*EPHB2*,*HAS2*,*MYB*,*NTS*,*PDPN*,*PLAU*,*ROR1*,*THBD*,*TIAM1*
		11	Organization of cytoskeleton	−2.861	0.002	Inhibited	*BMP7*,*EPHB2*,*HAS2*,*MYB*,*NTS*,*PDPN*,*PLAU*,*RASSF5*,*ROR1*,*THBD*,*TIAM1*
	Organismal development	8	Size of body	−2.781	0.002	Inhibited	*BMP7*,*CXCL14*,*ITGA11*,*PLAU*,*SFRP4*,*SMPD3*,*TH*,*WNT2*
CHAR	Infectious diseases	5	Infection of mammalia	2.190	0.000	Activated	*ASGR1*,*CAMP*,*IGHM*,*PLAU*,*STX11*
KC	Energy production	6	Consumption of oxygen	2.449	0.004	Activated	*ETFBKMT*,*G0S2*,*LEPR*,*SERPINE1*,*SLC25A25*,*SLC2A1*
	Cell death and survival, embryonic development	5	Apoptosis of embryonic cells	2.236	0.001	Activated	*ACVR1C*,*ALX3*,*DOT1L*,*ITGA5*,*SLC2A1*
	Endocrine system development and function, molecular transport, small molecule biochemistry	10	Concentration of hormone	−2.146	0.005	Inhibited	*DUOXA2*,*FSTL3*,*LDLR*,*LEPR*,*PDK4*,*SMPD3*,*TLR5*,*TNFRSF12A*,*TTR*,*WNK4*
	Connective tissue development and function, tissue morphology	20	Quantity of connective tissue	−2.298	0.000	Inhibited	*CIDEA*,*CLEC10A*,*CXCL8*,*DLK1*,*FSTL3*,*FZD9*,*G0S2*,*LDLR*,*LEPR*,*MFSD2A*,*NFATC2*,*PDK4*,*SERPINE1*,*SLC20A1*,*SLC25A25*,*SLC7A1*,*SOST*,*SREBF1*,*TLR5*,*TNFSF10*
	Lipid metabolism, molecular transport, small molecule biochemistry	11	Concentration of triacylglycerol	−2.319	0.000	Inhibited	*ACLY*,*CIDEA*,*DLK1*,*G0S2*,*LDLR*,*LEPR*,*MFSD2A*,*PDK4*,*SERPINE1*,*SREBF1*,*TLR5*
	Nutritional disease	17	Obesity	−2.531	0.000	Inhibited	*CCRL2*,*CHRNA1*,*CIDEA*,*CXCL8*,*DLK1*,*G0S2*,*LDLR*,*LEPR*,*MFSD2A*,*PDK4*,*PROX1*,*SCN11A*,*SCN3A*,*SERPINE1*,*SLC25A25*,*SREBF1*,*TLR5*

^†^z-score ≥ 2 and z-score ≤ − 2 indicate Activated and Inhibited activation state.

**Table 5:** Predicted function of differentially expressed (DE) genes in subcutaneous adipose tissue of involved in the predicted lipid metabolic function using IPA.

Predicted functions	z-score^†^	DE gene*	Gene description	log_2_FC^#^	Adjusted P value	Location
Concentration of triacylglycerol (KC)	−2.319	*DLK1*	delta like non-canonical Notch ligand 1	2.474	0.002	Plasma Membrane
		*LEPR*	leptin receptor	2.057	0.024	Membrane
		*TLR5*	toll like receptor 5	1.617	0.012	Plasma Membrane
		*PDK4*	pyruvate dehydrogenase kinase 4	1.230	0.046	Cytoplasm
		*ACLY*	ATP citrate lyase	−1.364	0.045	Cytoplasm
		*CIDEA*	cell death-inducing DFFA-like effector a	−1.012	0.017	Cytoplasm
		*G0S2*	G0/G1 switch 2	−1.583	0.013	Cytoplasm
		*LDLR*	low density lipoprotein receptor	−1.653	< 0.001	Plasma Membrane
		*MFSD2A*	major facilitator superfamily domain containing 2A	−1.611	0.046	Plasma Membrane
		*SERPINE1*	serpin family E member 1	−1.464	0.001	Extracellular Space
		*SREBF1*	sterol regulatory element binding transcription factor 1	−1.140	< 0.001	Nucleus

^†^z-score ≥ 2 and z-score ≤ − 2 indicate Activated and Inhibited activation state.

*Differentially expressed genes were based on pairwise comparison between H- and L-RFIfat with FDR ≤ 0.1 and |log_2_FC|≥ 1 as cut-off.

^#^Log_2_FC ≥ 1 and log_2_FC ≤  − 1 indicate up- and down-regulation of a gene in L-RFIfat compared to H-RFIfat steers, respectively.

### Correlations between DE genes and FAs profiles

**Figure 4:**
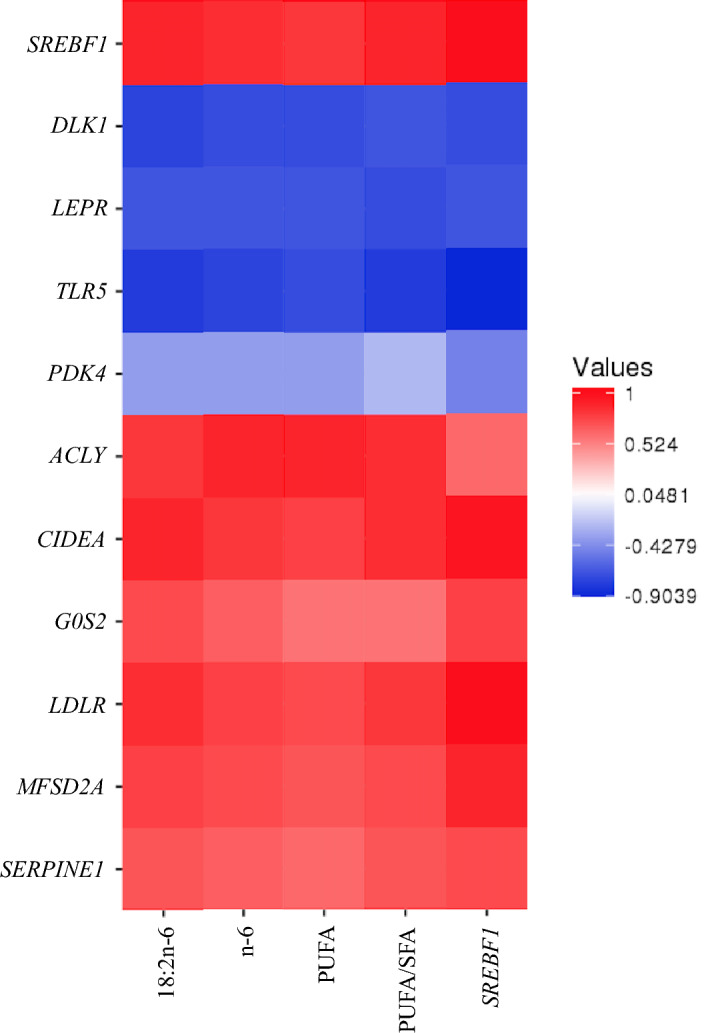
Spearman correlation analysis among the DE gene reads, and between the DE gene reads and FA composition. The correlation coefficient is colored by different intensities of red (positive correlation) and blue (negative correlation). DLK1 = delta like non-canonical Notch ligand 1; LEPR = leptin receptor; TLR5 = toll like receptor 5; PDK4 = pyruvate dehydrogenase kinase 4; ACLY = ATP citrate lyase; CIDEA = cell death-inducing DFFA-like effector a; G0S2 = G0/G1 switch 2; LDLR = low density lipoprotein receptor; MFSD2A = major facilitator superfamily domain containing 2A; SERPINE1 = serpin family E member 1; SREBF1 = sterol regulatory element binding transcription factor 1; n-6 (sum of omega 6 fatty acids) = 18:2n-6 + 20:3n-6 + 20:4n-6; PUFA (sum of polyunsaturated fatty acid) = 18:2n-6 + 20:3n-6 + 20:4n-6 + 18:3n-3; PUFA/SFA = ratio of PUFA to SFA.

When the relationships between expression of DE genes and FA profiles were further analyzed, correlation was only identified for KC steers. Proportion of C18:2n-6, total n-6, PUFA and PUFA/SFA were negatively correlated with expression of the up-regulated DE genes (*DLK1*,* LEPR*,* TLR5*,* PDK4*) (r values ranged from − 0.81 to − 0.57, except for the low value between PDK4 and PUFA/SFA) and positively correlated with the expression of down-regulated DE genes (*ACLY*,* CIDEA*,* G0S2*,* LDLR*,* MFSD2A*,* SERPINE1*,* SREBF1*) (r values ranged from 0.62 to 0.91). Meanwhile, expression of *SREBF1* was negatively correlated with the above up-regulated genes (r values ranged from − 0.90 to − 0.62), and positively correlated with the down-regulated genes (r values ranged from 0.69 to 0.98) (Fig. 4). The proposed mechanism through which proportions of these FAs being regulated by the genes is presented in Fig. 4.

## Discussion

The FAs in meat are key factors influencing product quality as more healthy FA profiles of beef products such as lowered SFA and higher n-3 FAs have been long pursued owing to their association with human health^28^. In past studies, the n-6/n-3 ratio has been used as the major indicator for the impact of the meat product on human health^29^. While it is not clear whether all FA weighted the same in terms of affecting human’s health, it may be more appropriate to consider all beneficial and all unhealthy FAs as a whole rather than only focus on a single component. Owing to the nature of the samples and the detection limit, the identified beneficial and unhealthy FAs, especially those with minor proportions, may be different across studies. Therefore, we have introduced the concept of beneficial/non-beneficial FA ratio (defined as healthy FA index) that expanded from the traditional n−6/n−3 ratio. In fact, the healthy FA index and the n-6/n-3 ratio were highly correlated (r = −0.50, *p* < 0.001) from our dataset, suggesting the potential application of this healthy FA index defined in this study as an alternate indicator of health impact of meat products. It should be noted that the component FAs for calculating the healthy FA index are modulable as a few commonly reported long-chain n-3 PUFAs were not detected due to their low proportion for the current sample set. For future usage of this concept, any detectable beneficial FAs and non-beneficial FAs should all be included for calculating the healthy FA index.

It is known that body fats are partly derived from the diet, partly derived from rumen microbial synthesis, and partly derived from endogenous synthesis, with dietary effect was more evident in affecting FA profiles in cattle tissues^30^ as well as influencing rumen microbial activities^31^. One of the limitations of the current study lies in the lack of dietary lipid component. In addition, as the animals were of different feed efficiency, their feed intake also affects the total dietary lipid to be consumed and may further affect the rumen microbial and cattle lipid metabolism and synthesis. Therefore, future study to include the dietary lipid composition combined with the animal intake should be taken into account to determine the origins of the FAs. However, as the main purpose of the current study was to identify whether animals of different breeds would have different regulatory mechanisms in its FA profiles, with the identical diet for all three breeds of animals, we were still able to identify the breed effect on FA and gene expression profiles in the subcutaneous fat tissue.

It has been widely reported that cattle of different breeds exhibit distinctive fatty acids related phenotypes as a result of their genetic variations. Our results showed that both subcutaneous adipose tissue thickness and FA profiles differed among the three breeds as expected. Our results showed that subcutaneous adipose tissue thickness of KC and ANG were approximately 35% higher than that of CHAR. Similar results were also reported by Mukiibi^32^ who compared the subcutaneous adipose tissue thickness among a large beef cattle herd composed of the same three breeds, as well as in the study by William^33^ when comparing the subcutaneous adipose tissue thickness between Angus × Hereford and CHAR cattle, suggesting a strong breed effect on subcutaneous adipose tissue thickness. Both KC and ANG belong to the small-size early maturing breeds, while CHAR belongs to the large-size late maturing breed^34^. Subcutaneous adipose tissue has been reported to be the last tissue to mature^35^, which may explain the thinner subcutaneous adipose tissue measured from CHAR compared to KC and ANG.

When analyzing the FA profiles among the entire dataset, the predominant FAs identified from subcutaneous adipose tissue were C16:0 and C18:0 as SFA, *c*9-18:1 as MUFA and 18:2n-6 as PUFA, which was comparable with the previous studies^36–38^. Breed effects on subcutaneous adipose tissue FA profiles have been widely reported for cattle. For instance, Dance et al.^39^ found 9 out of the 10 measured subcutaneous adipose tissue FA differed among five cattle breeds (Longhorn, Charolais cross, Hereford, Belted Galloway and Beef Shorthorn); Barton et al.^40^ found 7 out of the 10 measured FAs differed between Charolais and Limousin heifers; while Gamarra et al.^41^ reported higher MUFA and CLA contents in subcutaneous adipose tissue in Pirenaica heifers compared to Salers cattle and Holstein–Friesian cull cows. Comparable results were also observed in the current study for both individual FAs and grouped FA traits, where 37 out of the identified 49 measurements differed among breeds. The FAs for ANG and CHAR steers fell in the range as previously reported for different Angus herds^41,42^ and CHAR herds^43^. The KC steers which were produced from crosses between Angus, Charolais or Alberta Composite bulls and University of Alberta Composite dam line^44^, showed uniquely high values in beneficial FAs (*c*9-18:1, CLA, *t*11, *t*13-18:2) and healthy FA index compared to ANG and CHAR steers, suggesting that beef products from KC may have more favorable FA profiles compared to ANG and CHAR breeds. Recently Zhang et al.^38^ have examined the heritability of individual FAs in subcutaneous adipose tissue of a large beef cattle population (N = 1,366) belonging to six different genetic backgrounds using two different matrices, and only identified weak to moderate heritability of 25 major FAs. Regardless the differences in the examined animal breeds between our study and Zhang et al.^38^, significant variation among cattle breeds were also identified for most of these 25 FAs, suggesting that host genetics plays an essential role in regulating its subcutaneous adipose tissue FA profiles. Similar to individual FAs, beneficial FA indicators n-6:n-3 ratio which indicates the percentage of beneficial FAs was found to be different across different breeds^45^. Comparably, the measured healthy FA index also differed among the three breeds in the current study. According to Akanno et al.^46^, the KC breed was strongly influenced by Hereford (approximately 50%), a breed which was reported to have high CLA component in its fat tissue^47^. Although the exact percentage of the host genetic effects on individual FA and beneficial FA contents has not been dissected, we speculate that breed selection (such as the hybrid KC steers) may help to increase the content of beneficial FAs and thus further enhanced the healthy FA index compared to ANG and CHAR cattle.

Lipogenesis and fat partitioning are essential processes that are reported to be linked to cattle energetic efficiency^48^. They compared gene expression in subcutaneous adipose tissue between high and low RFI bulls and heifers and found that low RFI cattle exhibit upregulation of glucose metabolism in adipose tissue. However, gene expression was not linked to FA profiles, and it was not clear whether such variations in gene expression applies to different breeds and herds. The off-test subcutaneous adipose tissue thickness was reported to be responsible for 2%–5% of variation in feed intake, and RFI adjusting for off-test subcutaneous adipose tissue thickness makes RFI independent of body composition in feeder cattle^23^ and body fatness in replacement heifers^23^. As such, in the current study, the RFI values of the steers were all adjusted for subcutaneous adipose tissue thickness, and the FA profiles were compared between efficient (L-RFIfat) and inefficient (H-RFIfat) for each breed respectively. Surprisingly, in ANG and CHAR steers, none of the FAs exhibited linear correlations with RFIfat or RFI (data not shown), and most of the measured FAs did not differ between H-RFIfat and L-RFIfat groups, suggesting that selection for RFIfat did not have significant impact on the lipid metabolisms of subcutaneous adipose tissue in these two breeds. In contrast, RFIfat influenced the subcutaneous adipose tissue FA profiles of KC steers, where the amount of five FA differed between H-RFIfat and L-RFIfat groups, and healthy FA index tended to be higher in H-RFIfat KC steers (Table 3). Although the RFIfat was used as the main feed efficiency indicator, we performed additional analyses using the RFI only classification. As shown in Table S5, the results were comparable with that shown in Table 3, where variation between H- and L-RFI was only observed for KC animals. The effect of RFI on subcutaneous adipose tissue FAs was also reported for Nellore bulls, that the subcutaneous adipose tissue of H-RFI bulls contained higher cis vaccenic FA and lower oleic FA compared to that in L-RFI bulls^49^. Such opposing results may be due to the unique genetics of each cattle breed, as the markers for RFI were known to be breed-specific (Higgins et al., 2019). Among the three breeds involved in the current study, there may exist certain associations in the RFI markers and subcutaneous adipose tissue lipid metabolism markers for the KC cattle, which requires additional validation.

Fat deposition and FA profiles of the subcutaneous adipose tissue are regulated through complex regulatory mechanisms^41^. Past efforts to study the molecular mechanisms regulating the lipid metabolism and FA profiles largely rely on microarray and/or quantitative reverse transcription PCR to reveal the gene expression profiles of the subcutaneous adipose tissue^50,51^. The past methods largely relied on the known genes or the pre-designed chips, which limited the molecules being studied. With the advances in sequencing methods, RNA-Seq based transcriptome analyses which covers all expressed genes within the sample, has been applied recently in the studies of bovine subcutaneous adipose tissue for different breeds of cattle globally^52–54^. The transcriptome including the mapped reads, the identified gene numbers and the enriched GO terms of adipose tissues were comparable to those reported in Sun et al.^54^ in which a larger animal population including the same three breeds of steers were examined. The commonly expressed genes among the three breeds were also similar to previous studies^52–54^, suggesting that the core FA metabolism of subcutaneous adipose tissue were similar regardless of the varied animal breeds and feed regimes. Beyond the commonly expressed genes, a number of genes exclusively expressed in the subcutaneous adipose tissue of KC steers were significantly higher than ANG and CHAR (160 vs. 48 vs. 77). Among the breed-specific genes, *STAR* (Steroidogenic acute regulatory protein) was only expressed in the subcutaneous adipose tissue of KC steers. The product of *STAR* gene, *StAR* protein, controlled the rate-limiting step in steroidogenesis^55^, and *STAR* was found to trigger cholesterol delivery to the inner mitochondrial membrane thereafter initiate steroidogenesis in adipose tissue depots in Holstein cows^56^. As KC contains a Holstein component at approximately 20%^44^, the expression of *STAR* may reflect the genetic impact of Holstein in the KC breed and it may also explain the higher subcutaneous adipose tissue thickness observed for the KC steers. Although other breed-specific genes were not directly involved in lipid metabolism, their functions in other biological processes may indirectly influence the subcutaneous adipose tissue FA profiles, which quires further validation.

To illustrate the potential molecular mechanisms for the varied FA profiles between different RFI groups, the transcriptome was then compared between H-RFI and L-RFI steers for each breed respectively owing to the uniqueness of the transcriptome among the three breeds as reported above. Among the identified DE genes, those directly involving metabolism may explain the variations in the FA profiles observed among the three breeds. Carbohydrate metabolism related genes *BMP7* (bone morphogenetic protein 7) and *GFPT2* (glutamine-fructose-6-phosphate transaminase 2) were down-regulated in L-RFI ANG steers (Supplementary Table S3). *BMP7* was reported to be associated with subcutaneous adipose tissue thickness, carcass marbling score^57^ and feed efficiency traits^58^ across six cattle breeds (Angus, Charolais, Kinsella Composite, Elora crossbred, PG1, and TXX) through GWAS screening; while *GFPT2* acts as a catalytic enzyme in the hexosamine biosynthetic pathway (part of glucose metabolism) and generates UDP-N-acetyl-d-glucosamine the donor substrate for glycosylation reactions^59^. The lower expression of *BMP7* and *GFPT2* in L-RFI ANG steers suggests that the corresponding carbohydrate metabolism may be less active in efficient animals compared to inefficient animals. As carbohydrate metabolism products such as acetyl CoA can serve as the substrates for lipid synthesis, it can be speculated that the reduced carbohydrate metabolisms may further limit lipid metabolism in L-RFI ANG steers. This is in accordance with the study by Alexandre et al.^60^, who found higher expression of genes associated with lipid synthesis and higher deposition of subcutaneous adipose tissue in less feed efficient animals.

When considering the lipid metabolism-related genes, sterol regulatory element binding transcription factor 1 (*SREBF1*) and another 10 genes (*DLK1*, *CIDEA*, *LDLR*, *LEPR*,* TLR5*,* PDK4*,* ACLY*,* G0S2*,* MFSD2A*, and *SERPINE1*) that are involved in reducing triacylglycerol concentration were found differentially expressed between H- and L-RFI steers in KC cattle. Functions of these genes have been reported in other studies using mice models. Overexpression of *CIDEA* (cell death-inducing DFFA-like effector) in mouse hepatocytes promoted lipid accumulation and triacylglycerol (triglyceride) storage; knockdown of *CIDEA* adversely affected the ability of *SREBF1* to stimulate lipid accumulation^61^. *LDLR* (low density lipoprotein receptor) was involved in lipoprotein trafficking, playing a physiological role in maintaining cholesterol homeostasis, essential energy production, cell membrane, and hormone synthesis^62^; whereas *DLK1* (delta like non-canonical Notch ligand 1) induced negative feedback regulation in adipogenesis potential of murine 3T3-L1 cells^63^. However, whether these genes also function similarly in cattle adipose tissue needs further investigation. Being an upstream regulator of *DLK1*/*CIDEA*/*LDLR/ACLY*, the downregulation of *SREBF1* in L-RFI KC steers can be directly linked to the upregulation of *DLK1* and the downregulation of *CIDEA/LDLR/ACLY*, which further contribute to the lowered 18:2n-6, n-6, PUFA, and PUFA/SFA observed for the L-RFI KC steers (Fig. 4). However, this may also due to the upregulation of endogenous fatty acid synthesis in KC, and dilution of PUFA and their biohydrogenation products. It should be noted that, although these beneficial FAs were lower in L-RFI KC steers, KC still exhibited the highest healthy FA index among the three breeds. It was reported that *SREBF1* expression was associated with marbling score in Angus bulls^64^, and marbling was higher in inefficient cattle^65^. Therefore, *SREBF1* may be used as a marker for carcass traits although the gene expression in intramuscular fat was not measured in the current study. Combining these data, future studies promoting the *SREBF1* gene in KC steers may have a positive effect by triggering opposing effects on these downstream DE genes, thus further enhancing the beneficial FAs and/or marbling of the KC steers. Selectively breeding cattle based on the SNPs identified from *SREBP1* may be another option to increase the proportion of healthy FAs KC steers. In Korean Hanwoo cattle, SNPs of *SREBP1* gene had significant effect on marbling score, MUFA and *c*9-18:1^66^. It would also worth exploring the associations between these key genes and the RFI-indicating SNPs. If markers having dual-indicating potential for both host feed efficiency and beneficial FA profiles can be identified, it will magnify the industry benefits.

One of the possibilities that the RFIfat-associated variation in FA profiles and expression of genes was only observed for KC steers may be due to the more comprehensive pedigree among the herd. These could be due to the masked animal effects (sire/dam). We therefore further analyzed the sire/dam information of the animals, aiming to explore whether sire/dam effects may have influenced the measured parameters. Among all the KC steers, the sire information of 11 animals were unknown while the rest we the offspring of 18 sires and 45 dams; all the ANG steers were the offspring of 12 sires and 44 dams; and all the CHAR steers were the offspring of 13 sires and 40 dams. Therefore, it is not surprising that more DE genes and varied FA proportions were observed for KC than ANG and CHAR as more sires were included. However, we were unable to analyze the sire effect in the current study as some sires and almost all dams only had one offspring to be included in this study. It is worth to extend the analyses to a larger herd, with multiple offspring of the same sire/dam included, which may allow us to analyze the genetic effect on the FA profiles and gene expressions more precisely.

## Conclusions

FA profiles and healthy FA index in subcutaneous adipose tissue differed among three cattle breeds in the current study. KC and CHAR cattle had higher beneficial FAs and healthy FA index than ANG, which may be explained by the unique transcriptome of the adipose tissue among the three breeds. Effect of RFI classification on the FA profiles in subcutaneous adipose tissue of steers was breed dependent. Low RFI (high efficient) steers had the lower proportion of beneficial FAs such as 18:2n-6, total n-6, PUFA, and t11-18:1 and c9, t11-CLA, as well as the higher unhealthy FAs (i.e. 16:0) in subcutaneous tissue, especially within KC breed. Genetic selection for FA profiles that contributing to beef quality is breed-dependent. Key genes such as *SREBF1* may serve as the genetic markers for such selection, but validation of the potential marker genes in a larger population is needed.

## Methods

### Animals, experimental design, and sampling

Angus (ANG; n = 47), Charolais (CHAR; n = 48), and Kinsella Composite (KC; n = 48) steers, born and raised in either 2014 or 2015 at Roy Berg Kinsella Ranch, University of Alberta (Alberta, Canada) were used for this study. The Angus and Charolais herds were mated via artificial insemination, and live clean-up bulls registered by the Canadian Angus and Charolais Associations, respectively^58^. The Kinsella Composite herd was produced from crosses between Angus, Charolais, or Alberta Composite bulls and the University of Alberta’s Composite dam line^58^, which contains approximately 50% Hereford and 30% Angus breeds with 20% infusion of Holstein^44^. Steers were housed in a feedlot and fed a finishing diet (75% barley grain, 20% barley silage, and 5% pellet supplement that included Rumensin™ (as fed, 76.5% dry matter; 14.7% crude protein, 18.3% acid detergent fibre, 32% neutral detergent fibre, 1.2% calcium, 0.45% phosphorus, and 70% total digestible nutrients on a dry matter basis; 0.24 ppm magnesium, 0.93 ppm potassium, 0.28 ppm sodium, 604 ppm iron, 128 ppm manganese, 182 ppm zinc, and 29.4 ppm copper) for three months. Feed intake was recorded using the GrowSafe feed intake monitoring system (GrowSafe Systems Ltd., Airdrie, Alberta, Canada) and body weight was measured every 14 days to calculate average daily gain (ADG). Residual feed intake adjusted for subcutaneous adipose tissue thickness was calculated based on procedures reported by Basarab et al.^23^. In addition, the marbling score of each steer was obtained from Jiu et al. (2019). Subcutaneous adipose tissue was collected within 30 min after slaughter, snap-frozen in liquid nitrogen, and subsequently stored at − 80 °C until RNA extraction and FA analysis.

### Fatty acid analysis

Subcutaneous adipose tissue (50 mg) was weighed and freeze-dried^15^. One ml of 1 mg c10-17:1 methyl ester/ml hexane (standard no. U-42 M from Nu-Check Prep Inc., Elysian, MN, USA), the internal standard, was added to freeze-dried samples followed by 2 ml of 0.5 M sodium methoxide and direct methylated for 15 min at 50 °C^66^, cooled, 1 ml water and 1 ml hexane added, shaken, and fatty acid methyl esters (FAME) were collected in the hexane layer. FAME were analyzed with a CP-3800 gas chromatograph (GC, Varian Inc., Walnut Creek, CA): most FAME were analyzed following the protocol of Kramer et al.^67^ using a CP-Sil88 column (100 m, 25 µm ID, 0.2 µm film thickness, Agilent Technologies, Santa Clara, CA); while an SLB IL 111 column (30 m, 0.25 mm ID, 0.2 mm film thickness, Supelco Inc., Bellefonte, PA) was used to measure t7,c9-18:2 and c9,t11-18:2 according to Turner et al.^68^. Reference standard no. 601 (Nu-Chek Prep Inc., Elysian, MN, USA) was used to identify most of the FAME; reference standard BC-Mix1 (Applied Science, State College, PA, USA) was used to identify branched-chain FAME; while the UC-59 M standard (Nu-Chek Prep Inc.) contained all four positional CLA isomers was used for detection of CLA isomers. For FAs not included in the standard mixtures such as several trans-18:1, CLA and other biohydrogenation intermediates, these were identified by their retention times and elution orders according to previous studies^15,17,69^. Quantification of FAME was calculated using chromatographic peak area and internal standard following Vahmani et al.^70^. The beneficial/non-beneficial fatty acid ratio (termed as healthy FA index) was defined as the total proportion of beneficial FAs (18:3n-3; c9,t11-18:2 and t11-18:1)/total proportion of non-beneficial FAs (C14:0; C16:0; and t10-18:1). The effect of RFI on FA profiles were compared between high RFI (H-RFI, RFI > 0.5, low feed efficiency) and low RFI (L-RFI, RFI < − 0.5 high feed efficiency) steers within each breed.

### RNA extraction and sequencing

Total RNA was extracted from the samples according to Sun et al*.*^71^. Briefly, the subcutaneous adipose tissue was ground with liquid nitrogen using a frozen mortar and pestle prior to RNA extraction. Total RNA was extracted from 100 mg of ground subcutaneous adipose tissue using RNeasy Lipid Tissue Mini Kit (Qiagen, Germany) according to the manufacturer’s instructions. The quantity and quality of the RNA were assessed using Qubit 2.0 Fluorometer (Invitrogen, Carlsbad, CA) and the Agilent 2200 TapeStation (Agilent Technologies, Inc., Santa Clara, USA), respectively. The samples with RNA integrity number (RIN) > 7.0 were used for RNA-Seq library construction.

Due to the limited amount and lipid rich characteristics of the samples, as well as to examine the animals with extreme RNA classification, only RNA extracted from 22 samples (KC = 8; AN = 8; CH = 6; with top or bottom RFI values for each breed) passed the criteria described above and were subjected to RNA-Seq library construction. The total RNA (1 µg) from each sample was first treated with Ribo-Zero Gold Kit (Illumina, San Diego, CA, USA) to remove ribosomal RNA. The rRNA-free RNA samples were then used to construct cDNA libraries according to the protocol of TruSeq Stranded Total RNA Sample Prep Kit (Illumina, San Diego, CA, USA). After quantification with a Qubit 2.0 Fluorometer (Invitrogen, Carlsbad, CA, USA), cDNA libraries with effective concentration of 2 nM were pooled and sequenced (100 bp paired-end) using the Illumina HiSeq™ 4000 platform at the Genome Quebec Innovation Centre, McGill University (Montreal, Quebec, Canada).

### Transcriptome data analysis

RNA-Seq data analysis was performed according to the pipeline reported previously^72^. Briefly, adapter sequences were removed, and the quality of reads was filtered using fastq-mcf with quality score ≥ 20 and read length ≥ 75^73^. The clean reads were then aligned to bovine genome UMD3.1 (http://bovinegenome.org/?q=umd_downloads, accessed on May 4, 2017) and assembled by TopHat2 software package (v2.0.9) with default parameters^74,75^. Samtools (v1.1)^76^ was used to sort the BAM alignment files and then convert them to SAM format. Then, the number of reads mapped to each gene was counted by HTSeq-count (v0.6.1)^77^. The expression level of mRNA in tissue sample was calculated by normalizing reads to counts per million (CPM) using the following formula: CPM = (gene counts number/total mapped counts number) × 10^6^. Only genes with CPM > 1 were considered as being expressed (Wilkinson et al., 2016). Only genes expressed in at least 50% of animals within each breed (KC ≥ 4, AN ≥ 4, CH ≥ 3) were considered in the DE gene analysis. Analysis of differentially expressed (DE) genes was performed using Bioconductor package edgeR (v3.4.1)^78^. An absolute value of log_2_ fold change (log_2_FC) ≥ 1 together with a false discovery rate (FDR) adjusted by Benjamini–Hochberg method^79^ < 0.1 was used as cut-off to determine significantly DE genes^80^, where log_2_FC > 1 was considered as up-regulated and log_2_FC < − 1 was considered as down-regulated. All data transformation and filter criteria followed Kong et al.^81^.

### Functional analysis of common and differentially expressed genes

The functional analysis of commonly expressed genes and DE genes between H- and L-RFI animals was performed using several bioinformatics tools. Commonly expressed genes were those identified in samples from all animals. Ensembl gene ID lists were converted to gene symbols using the Database for Annotation, Visualization, and Integrated Discovery (DAVID; https://david.ncifcrf.gov) and Ingenuity Pathway Analysis (IPA; https://www.qiagenbioinformatics.com). To increase the resolution of functional profiles of the transcriptome, multiple tools including PANTHER (Protein Analysis Through Evolutionary Relationships (http://pantherdb.org)) classification system^82^, DAVID and IPA were used to perform the functional classification and enrichment analysis based on Gene Ontology (GO) annotations. In addition, upstream regulator analysis was performed to identify the biological influence on DE genes using IPA.

### Statistical analysis

Effects of breed and RFI on FA profiles were identified using PROC MIXED of SAS (version 9.2; SAS Institute, Inc., Cary, NC, USA). The statistical model was Y_ij_ = μ + B_i_ + R_j_ + (B × R)_ij_ + e_ij_, where Y_ij_ was the response variable, μ was the overall mean, B_i_ was the fixed effect of breed (i = CH, AN, KC), R_j_ was the fixed effect of RFI (j = high, low), (B × R)_ij_ was the interaction of breed with RFI, and e_ij_ was the random residual error. Individual animal was included as a random factor. For analysis within each breed, B_i_ and (B × R)_ij_ were removed from the model. Significant differences between LS-Means of treatments were assessed by the PDIFF option. Spearman correlation was analyzed using PROC CORR procedure (SAS 9.2) to investigate the relationships between the parameters, such as RFI values and FA proportions, CPMs of the DE genes and FA proportions, as well as among the identified DE genes. A p-value of < 0.05 was used to designate significant differences for the MIXED models and correlations, while those p-values between 0.05 and 0.1 were considered as trends.

### Ethics approval

This study was performed in compliance of ARRIVE guidelines. All experimental procedures were managed in accordance with the guidelines for animal care provided by Canadian Council of Animal Care^83^ and the animal trial protocol was approved by Animal Care and Use Committee, University of Alberta (Protocol number AUP00000882).

### Sequencing data availability

All RNA-Seq data were deposited in National Center for Biotechnology Information (NCBI) Gene Expression Omnibus (GEO) database with accession number GSE107268.

## Supplementary Information

Supplementary Information 1.

Supplementary Information 2.

Supplementary Information 3.

Supplementary Information 4.

Supplementary Information 5.

Supplementary Information 6.

Supplementary Information 7.

Supplementary Information 8.
