# Supplementary material for: Breed dependent regulatory mechanisms of beneficial and non-beneficial fatty acid profiles in subcutaneous adipose tissue in cattle with divergent feed efficiency
Source: Sci Rep. 2022 Mar 17;12:4612. doi: 10.1038/s41598-022-08572-8 (PMC8931072; doi:10.1038/s41598-022-08572-8)
Supplement: Supplementary file 1 — Supplementary Information 1. [file 41598_2022_8572_MOESM1_ESM.docx]

**Breed dependent regulatory mechanisms of beneficial and non-beneficial fatty acid profiles in subcutaneous adipose tissue in cattle with divergent feed efficiency**

Mi Zhou^a,#^, Zhi Zhu^b#^, Hui-Zeng Sun^c^, Ke Zhao^d^, Mike E.R. Dugan^e^, Heather Bruce^a^, Carolyn Fitzsimmons^a,e^, Changxi Li^a,e^, and Le Luo Guan^a^

**Supplementary Figure S1.** RNA-sequencing results. (A) Number of raw reads obtained from libraries. (B) Number of clean reads after quality control with quality score ≥ 20 and leangth ≥ 75. (C) Number of reads aligned to the reference bovine genome. (D) Number of reads aligned to bovine gene.
