# Supplementary material for: Breed dependent regulatory mechanisms of beneficial and non-beneficial fatty acid profiles in subcutaneous adipose tissue in cattle with divergent feed efficiency
Source: Sci Rep. 2022 Mar 17;12:4612. doi: 10.1038/s41598-022-08572-8 (PMC8931072; doi:10.1038/s41598-022-08572-8)
Supplement: Supplementary file 2 — Supplementary Information 2. [file 41598_2022_8572_MOESM2_ESM.docx]

**Breed dependent regulatory mechanisms of beneficial and non-beneficial fatty acid profiles in subcutaneous adipose tissue in cattle with divergent feed efficiency**

Mi Zhou^a,#^, Zhi Zhu^b#^, Hui-Zeng Sun^c^, Ke Zhao^d^, Mike E.R. Dugan^e^, Heather Bruce^a^, Carolyn Fitzsimmons^a,e^, Changxi Li^a,e^, and Le Luo Guan^a^

**Supplementary Figure S2.** Distribution of the identified differential expressed genes among the three breeds. Up-regulated and Down-regulated referred to the differential expressed genes between H-RFI and L-RFI animals.
