# Supplementary material for: Breed dependent regulatory mechanisms of beneficial and non-beneficial fatty acid profiles in subcutaneous adipose tissue in cattle with divergent feed efficiency
Source: Sci Rep. 2022 Mar 17;12:4612. doi: 10.1038/s41598-022-08572-8 (PMC8931072; doi:10.1038/s41598-022-08572-8)
Supplement: Supplementary file 7 — Supplementary Information 7. [file 41598_2022_8572_MOESM7_ESM.docx]

**Table S5.** Effects of breed and residual feed intake (RFI) on fatty acid proportion (%) in subcutaneous tissues and performance traits of beef cattle.

| Trait^*^ | ANG^1^ | | | CHAR^2^ | | | KC^3^ | | | SEM | *p* value | | |
| --- | --- | --- | --- | --- | --- | --- | --- | --- | --- | --- | --- | --- | --- |
|  | H-RFI^4^  (n = 16) | L-RFI^5^  (n = 14) | *p* value^#^ | H-RFI  (n = 17) | L-RFI  (n = 17) | *p* value^#^ | H-RFI  (n = 18) | L-RFI  (n = 21) | *p* value^#^ |  | Breed | RFI | Breed × RFI |
| 10:0 | 0.05^b^ | 0.06^b^ | 0.096 | 0.07^a^ | 0.06^b^ | 0.062 | 0.06^b^ | 0.06^b^ | 0.968 | 0.001 | 0.014 | 0.508 | 0.022 |
| 12:0 | 0.07^c^ | 0.08^bc^ | 0.255 | 0.11^a^ | 0.10^ab^ | 0.207 | 0.09^bc^ | 0.09^b^ | 0.608 | 0.002 | 0.0003 | 0.903 | 0.181 |
| 14:0 | 3.75^bc^ | 3.89^abc^ | 0.463 | 4.32^a^ | 4.13^ab^ | 0.426 | 3.66^c^ | 4.05^abc^ | 0.126 | 0.069 | 0.029 | 0.405 | 0.197 |
| 15:0 | 0.45^ab^ | 0.51^a^ | 0.275 | 0.51^a^ | 0.46^ab^ | 0.268 | 0.41^b^ | 0.45^ab^ | 0.238 | 0.012 | 0.110 | 0.544 | 0.168 |
| 16:0 | 28.07^a^ | 28.29^a^ | 0.769 | 27.01^ab^ | 26.16^b^ | 0.156 | 26.21^b^ | 27.67^a^ | 0.029 | 0.200 | 0.003 | 0.466 | 0.038 |
| 17:0 | 1.10^a^ | 1.13^a^ | 0.745 | 0.99^ab^ | 0.91^b^ | 0.258 | 0.92^b^ | 0.90^b^ | 0.698 | 0.025 | 0.002 | 0.624 | 0.613 |
| 18:0 | 12.13 | 11.56 | 0.566 | 11.98 | 11.85 | 0.863 | 11.02 | 10.81 | 0.791 | 0.240 | 0.159 | 0.534 | 0.933 |
| 19:0 | 0.19^ab^ | 0.19^ab^ | 0.835 | 0.21^ab^ | 0.23^a^ | 0.403 | 0.18^b^ | 0.19^b^ | 0.424 | 0.006 | 0.067 | 0.351 | 0.611 |
| 20:0 | 0.08 | 0.08 | 0.897 | 0.09 | 0.09 | 0.999 | 0.08 | 0.08 | 0.783 | 0.002 | 0.017 | 0.960 | 0.960 |
| SFA | 45.90^a^ | 45.79^a^ | 0.903 | 45.29^a^ | 43.99^ab^ | 0.204 | 42.64^b^ | 44.30^ab^ | 0.209 | 0.333 | 0.014 | 0.895 | 0.156 |
| *iso*-14:0 | 0.03 | 0.03 | 0.990 | 0.03 | 0.03 | 0.810 | 0.03 | 0.03 | 0.730 | 0.001 | 0.723 | 0.979 | 0.916 |
| *iso*-15:0 | 0.12^b^ | 0.13^ab^ | 0.443 | 0.15^a^ | 0.14^ab^ | 0.515 | 0.14^a^ | 0.14^a^ | 0.636 | 0.003 | 0.017 | 0.689 | 0.542 |
| *anteiso*-15:0 | 0.14^c^ | 0.16^bc^ | 0.374 | 0.20^a^ | 0.19^ab^ | 0.557 | 0.16^bc^ | 0.16^bc^ | 0.604 | 0.005 | 0.002 | 0.641 | 0.530 |
| *iso*-16:0 | 0.15^b^ | 0.16^ab^ | 0.471 | 0.19^a^ | 0.18^ab^ | 0.512 | 0.17^ab^ | 0.16^ab^ | 0.417 | 0.006 | 0.081 | 0.710 | 0.496 |
| *iso*-17:0 | 0.30^c^ | 0.32^bc^ | 0.392 | 0.37^a^ | 0.37^a^ | 0.941 | 0.34^b^ | 0.34^b^ | 0.862 | 0.005 | <.0001 | 0.566 | 0.690 |
| *anteiso*-17:0 | 0.47^bc^ | 0.52^b^ | 0.108 | 0.61^a^ | 0.59^a^ | 0.567 | 0.46^c^ | 0.44^c^ | 0.230 | 0.010 | <.0001 | 0.782 | 0.111 |
| *iso*-18:0 | 0.11 | 0.12 | 0.363 | 0.12 | 0.11 | 0.366 | 0.13 | 0.10 | 0.062 | 0.003 | 0.893 | 0.306 | 0.153 |
| BCFA | 1.32^c^ | 1.44^bc^ | 0.232 | 1.67^a^ | 1.61^ab^ | 0.549 | 1.43^c^ | 1.38^c^ | 0.496 | 0.028 | <.0001 | 0.928 | 0.299 |
| SFA + BCFA | 47.22^a^ | 47.23^a^ | 0.986 | 46.96^a^ | 45.60^ab^ | 0.207 | 44.06^b^ | 45.68^ab^ | 0.237 | 0.347 | 0.019 | 0.894 | 0.183 |
| cyclohexyl-17:0 | 0.03 | 0.04 | 0.834 | 0.04 | 0.04 | 0.861 | 0.04 | 0.04 | 1.000 | 0.001 | 0.362 | 0.970 | 0.950 |
| *c*9-14:1 | 1.44 | 1.57 | 0.429 | 1.58 | 1.50 | 0.633 | 1.64 | 1.76 | 0.508 | 0.049 | 0.208 | 0.566 | 0.628 |
| *c*7-16:1 | 0.14^c^ | 0.14^c^ | 0.843 | 0.17^ab^ | 0.16^b^ | 0.435 | 0.18^a^ | 0.17^ab^ | 0.654 | 0.003 | <.0001 | 0.406 | 0.905 |
| *c*9-16:1 | 4.84^c^ | 4.93^bc^ | 0.843 | 5.51^abc^ | 5.77^a^ | 0.523 | 5.39^abc^ | 5.66^ab^ | 0.457 | 0.116 | 0.025 | 0.370 | 0.936 |
| *c*11-16:1 | 0.30 | 0.31 | 0.615 | 0.30 | 0.29 | 0.747 | 0.33 | 0.33 | 0.880 | 0.009 | 0.300 | 0.844 | 0.854 |
| *c*9-17:1 | 0.90 | 0.93 | 0.808 | 0.85 | 0.86 | 0.857 | 0.86 | 0.83 | 0.766 | 0.029 | 0.549 | 0.936 | 0.906 |
| *c*9-18:1 | 38.01^ab^ | 37.69^ab^ | 0.720 | 36.25^b^ | 37.48^b^ | 0.162 | 39.29^a^ | 37.97^ab^ | 0.186 | 0.274 | 0.024 | 0.804 | 0.134 |
| *c*11-18:1 | 1.69 | 1.67 | 0.908 | 1.88 | 1.99 | 0.485 | 1.98 | 1.87 | 0.549 | 0.049 | 0.072 | 0.953 | 0.615 |
| *c*12-18:1 | 0.10^cd^ | 0.09^d^ | 0.488 | 0.16^a^ | 0.15^ab^ | 0.477 | 0.15^ab^ | 0.13^bc^ | 0.276 | 0.005 | <.0001 | 0.159 | 0.907 |
| *c*13-18:1 | 0.55^ab^ | 0.54^abc^ | 0.824 | 0.45^bc^ | 0.44^c^ | 0.795 | 0.58^a^ | 0.53^abc^ | 0.367 | 0.015 | 0.004 | 0.400 | 0.775 |
| *c*14-18:1 | 0.13^b^ | 0.12^b^ | 0.783 | 0.16^a^ | 0.16^a^ | 0.657 | 0.16^a^ | 0.16^a^ | 0.977 | 0.004 | 0.0004 | 0.644 | 0.925 |
| *c*9-20:1 | 0.09^d^ | 0.10^cd^ | 0.088 | 0.10^bc^ | 0.10^bc^ | 0.969 | 0.11^a^ | 0.11^ab^ | 0.445 | 0.002 | <.0001 | 0.573 | 0.236 |
| *c*11-20:1 | 0.25^ab^ | 0.25^ab^ | 0.833 | 0.23^ab^ | 0.25^ab^ | 0.365 | 0.26^a^ | 0.22^b^ | 0.047 | 0.005 | 0.602 | 0.535 | 0.064 |
| *t*6-18:1 | 0.18^c^ | 0.19^c^ | 0.744 | 0.24^ab^ | 0.25^a^ | 0.768 | 0.20^bc^ | 0.19^c^ | 0.489 | 0.007 | 0.001 | 0.941 | 0.746 |
| *t*9-18:1 | 0.22^b^ | 0.23^b^ | 0.883 | 0.27^a^ | 0.28^a^ | 0.683 | 0.23^b^ | 0.22^b^ | 0.227 | 0.005 | <.0001 | 0.943 | 0.600 |
| *t*10-18:1 | 1.11^ab^ | 1.09^ab^ | 0.913 | 1.29^a^ | 1.33^a^ | 0.804 | 1.06^ab^ | 0.95^b^ | 0.456 | 0.050 | 0.039 | 0.767 | 0.790 |
| *t*11-18:1 | 0.54^b^ | 0.60^ab^ | 0.412 | 0.70^a^ | 0.63^ab^ | 0.358 | 0.64^ab^ | 0.61^ab^ | 0.491 | 0.019 | 0.113 | 0.665 | 0.390 |
| *t*12-18:1 | 0.16^b^ | 0.15^b^ | 0.814 | 0.20^a^ | 0.20^a^ | 0.779 | 0.20^a^ | 0.19^ab^ | 0.603 | 0.006 | 0.002 | 0.552 | 0.975 |
| *t*13-/*t*14-18:1 | 0.28^b^ | 0.27^b^ | 0.725 | 0.38^a^ | 0.36^a^ | 0.481 | 0.30^b^ | 0.29^b^ | 0.558 | 0.009 | <.0001 | 0.339 | 0.949 |
| *trans*-18:1 | 2.48^c^ | 2.51^c^ | 0.889 | 3.08^a^ | 3.05^ab^ | 0.880 | 2.64^bc^ | 2.44^c^ | 0.337 | 0.069 | 0.001 | 0.604 | 0.744 |
| MUFA | 50.91^b^ | 50.86^b^ | 0.963 | 50.72^b^ | 52.21^ab^ | 0.169 | 53.56^a^ | 52.17^ab^ | 0.301 | 0.343 | 0.049 | 0.982 | 0.207 |
| *c*9,*t*14-/*c*9,*t*13-18:2 | 0.15^c^ | 0.15^c^ | 0.889 | 0.21^a^ | 0.22^a^ | 0.759 | 0.20^ab^ | 0.19^b^ | 0.234 | 0.004 | <.0001 | 0.898 | 0.666 |
| *c*9,*t*15-18:2 | 0.07^b^ | 0.07^b^ | 0.548 | 0.09^a^ | 0.09^a^ | 0.964 | 0.10^a^ | 0.09^a^ | 0.615 | 0.002 | <.0001 | 0.930 | 0.758 |
| *c*9,*t*12-18:2 | 0.05^c^ | 0.05^c^ | 0.953 | 0.08^a^ | 0.08^a^ | 0.642 | 0.06^b^ | 0.06^bc^ | 0.121 | 0.002 | <.0001 | 0.330 | 0.683 |
| *t*11,*c*15-18:2 | 0.05 | 0.05 | 0.880 | 0.05 | 0.05 | 0.704 | 0.06 | 0.06 | 0.809 | 0.002 | 0.542 | 0.916 | 0.916 |
| *c*9,*c*15-18:2 | 0.12^a^ | 0.12^a^ | 0.825 | 0.09^b^ | 0.09^b^ | 0.342 | 0.14^a^ | 0.13^a^ | 0.442 | 0.004 | <.0001 | 0.422 | 0.713 |
| ADFA | 0.44^b^ | 0.45^b^ | 0.822 | 0.54^a^ | 0.53^a^ | 0.798 | 0.55^a^ | 0.53^a^ | 0.364 | 0.010 | <.0001 | 0.607 | 0.757 |
| *t*7,*c*9-18:2 | 0.05^c^ | 0.06^bc^ | 0.786 | 0.07^a^ | 0.07^a^ | 0.780 | 0.07^ab^ | 0.06^abc^ | 0.304 | 0.002 | 0.003 | 0.918 | 0.643 |
| *c*9,*t*11-18:2 | 0.17^b^ | 0.17^b^ | 0.954 | 0.11^b^ | 0.14^b^ | 0.560 | 0.34^a^ | 0.31^a^ | 0.245 | 0.014 | <.0001 | 0.984 | 0.561 |
| *t*9,*c*11-18:2 | 0.02 | 0.02 | 0.632 | 0.02 | 0.02 | 0.178 | 0.02 | 0.02 | 0.217 | 0.001 | 0.385 | 0.954 | 0.155 |
| *t*11,*t*13-18:2 | 0.01^b^ | 0.01^b^ | 0.703 | 0.01^b^ | 0.01^ab^ | 0.119 | 0.01^ab^ | 0.02^a^ | 0.223 | 0.001 | 0.045 | 0.204 | 0.403 |
| *t*7,*t*9-/*t*10,*t*12-18:2 | 0.016^b^ | 0.020^ab^ | 0.153 | 0.021^ab^ | 0.021^a^ | 0.963 | 0.024^a^ | 0.024^a^ | 0.900 | 0.001 | 0.011 | 0.354 | 0.571 |
| CLA | 0.27^b^ | 0.28^b^ | 0.902 | 0.23^b^ | 0.27^b^ | 0.413 | 0.47^a^ | 0.43^a^ | 0.210 | 0.014 | <.0001 | 0.925 | 0.418 |
| 18:2n-6 | 0.91^c^ | 0.93^c^ | 0.730 | 1.25^a^ | 1.14^ab^ | 0.144 | 1.07^b^ | 0.92^c^ | 0.001 | 0.021 | <.0001 | 0.019 | 0.110 |
| 20:3n-6 | 0.06^abc^ | 0.05^bc^ | 0.486 | 0.07^a^ | 0.05^c^ | 0.004 | 0.07^a^ | 0.06^ab^ | 0.696 | 0.002 | 0.038 | 0.018 | 0.095 |
| 20:4n-6 | 0.04^c^ | 0.04^c^ | 0.689 | 0.05^abc^ | 0.04^bc^ | 0.351 | 0.05^a^ | 0.05^ab^ | 0.579 | 0.001 | 0.003 | 0.275 | 0.896 |
| n-6 | 1.01^c^ | 1.02^c^ | 0.798 | 1.36^a^ | 1.23^b^ | 0.097 | 1.19^b^ | 1.03^c^ | 0.002 | 0.022 | <.0001 | 0.012 | 0.122 |
| 18:3n-3 | 0.12^b^ | 0.12^b^ | 0.969 | 0.14^a^ | 0.12^ab^ | 0.107 | 0.13^ab^ | 0.12^b^ | 0.446 | 0.003 | 0.145 | 0.195 | 0.543 |
| PUFA | 1.13^c^ | 1.14^c^ | 0.828 | 1.51^a^ | 1.36^b^ | 0.082 | 1.32^b^ | 1.15^c^ | 0.003 | 0.023 | <.0001 | 0.013 | 0.140 |
| PUFA/SFA | 0.024^b^ | 0.025^b^ | 0.778 | 0.033^a^ | 0.031^a^ | 0.198 | 0.031^a^ | 0.026^b^ | 0.003 | 0.001 | <.0001 | 0.015 | 0.074 |
| n-6/n-3 | 9.06^b^ | 9.13^ab^ | 0.928 | 9.88^ab^ | 10.42^a^ | 0.435 | 9.77^ab^ | 8.83^b^ | 0.042 | 0.184 | 0.049 | 0.765 | 0.212 |
| FA ratio | 0.025^c^ | 0.027^bc^ | 0.512 | 0.030^bc^ | 0.029^bc^ | 0.687 | 0.036^a^ | 0.031^ab^ | 0.093 | 0.001 | 0.0004 | 0.479 | 0.288 |
| SCD proxy | 0.27^ab^ | 0.28^ab^ | 0.647 | 0.27^b^ | 0.26^b^ | 0.912 | 0.30^a^ | 0.30^a^ | 0.999 | 0.005 | 0.013 | 0.841 | 0.924 |
| DMI (kg) | 12.49^a^ | 10.73^c^ | 0.001 | 11.87^ab^ | 9.83^d^ | <.0001 | 11.29^bc^ | 9.24^d^ | <.0001 | 0.156 | <.0001 | <.0001 | 0.826 |
| ADG (kg) | 1.62^ab^ | 1.79^a^ | 0.132 | 1.65^ab^ | 1.62^ab^ | 0.661 | 1.54^b^ | 1.51^b^ | 0.762 | 0.027 | 0.021 | 0.465 | 0.206 |
| Backfat thickness (mm) | 11.13^a^ | 11.28^a^ | 0.899 | 8.06^b^ | 8.25^b^ | 0.830 | 10.74^a^ | 11.93^a^ | 0.271 | 0.332 | <.0001 | 0.397 | 0.720 |
| Marbling score | 433.33^a^ | 409.29^ab^ | 0.257 | 400.00^abc^ | 381.18^bc^ | 0.331 | 380.33^bc^ | 367.37^c^ | 0.370 | 5.520 | 0.002 | 0.078 | 0.912 |

^*^*c* = *cis*; *t* = *trans*; SFA (sum of saturated fatty acid) = 10:0 + 12:0 + 14:0 + 15:0 + 16:0 + 17:0 + 18:0 + 19:0 + 20:0; BCFA (sum of branched chain fatty acid) = *iso*-14:0 + *iso*-15:0 + *anteiso*-15:0 + *iso*-16:0 + *iso*-17:0 + *anteiso*-17:0 + *iso*-18:0; SFA+BCFA = sum of SFA and BCFA; *trans-*18:1 (sum of *trans-*18:1) = *t*6-18:1 + *t*9-18:1 + *t*10-18:1 + *t*11-18:1 + *t*12-18:1 + *t*13-/*t*14-18:1; MUFA (sum of monounsaturated fatty acid) = *c*9-14:1 + *c*7-16:1 + *c*9-16:1 + *c*11-16:1 + *c*9-17:1 + *c*9-18:1 + *c*11-18:1 + *c*12-18:1 + *c*13-18:1 + *c*14-18:1 + *t*6-18:1 + *t*9-18:1 + *t*10-18:1 + *t*11-18:1 + *t*12-18:1 + *t*13-/*t*14-18:1 + *c*9-20:1 + *c*11-20:1; ADFA (sum of atypical dienes fatty acid) = *c*9,*t*14-/*c*9,*t*13-18:2 + *c*9,*t*15-18:2 + *c*9,*t*12-18:2 + *t*11,*c*15-18:2 + *c*9,*c*15-18:2; CLA (sum of conjugated linoleic acid) = *t*7,*c*9-18:2 + *c*9,*t*11-18:2 + *t*9,*c*11-18:2 + *t*11,*t*13-18:2 + *t*7,*t*9-/*t*10,*t*12-18:2; n-6 (sum of omega 6 fatty acids) = 18:2n-6 + 20:3n-6 + 20:4n-6; n-3 (sum of omega 3 fatty acids) = 18:3n-3; PUFA (sum of polyunsaturated fatty acid) = 18:2n-6 + 20:3n-6 + 20:4n-6 + 18:3n-3; PUFA/SFA = ratio of PUFA to SFA; n-6/n-3 = ratio of n−6 to n−3 PUFA; FA ratio = (18:3n-3 + *c*9,*t*11-18:2 + *t*11-18:1)/(14:0 + 16:0 + *t*10-18:1); *SCD* proxy (stearoyl-CoA desaturase proxy) = *c*9-14:1/(*c*9-14:1 + 14:0); DMI = dry matter intake; ADG = average daily gain.

^#^Statistical analysis was performed within each breed.

^1^Angus breed; ^2^Charolais breed; ^3^Kinsella Composite breed; ^4^High RFIfat steers (RFIfat > 0.5); ^5^Low RFIfat steers (RFIfat < -0.5).

Means in the same row with different superscripts (a, b, c, d) indicate significance (*p* < 0.05).
